# The Stress-Buffering Effect of Daily Physical Activity on Negative and Positive Affect

**DOI:** 10.1093/geroni/igaa057.1304

**Published:** 2020-12-16

**Authors:** Sun Ah Lee, Susanna Joo, Kayeon Lee, Hye Won Chai, Hey Jung Jun, David Almeida

**Affiliations:** 1 Yonsei University, Seoul, Republic of Korea; 2 The Pennsylvania State University, State College, Pennsylvania, United States

## Abstract

Previous studies show that physical activity is beneficial for emotional well-being. This study extends prior research by examining whether engagement in physical activity moderates the association between daily stressor severity and daily emotional well-being. We used data from the second wave of the National Study of Daily Experiences, a sub-project of the Midlife in the United States (MIDUS) study. Respondents (N = 1,851; ages 33 to84) reported their daily experiences across eight consecutive days. Multilevel models explored concurrent and lagged interaction effects between daily stressor severity and physical activity on negative and positive affect and whether these associations differed by age. Physical activity was measured by engagement in vigorous physical activity for at least 30 minutes. Results showed significant interactions between stressor severity and physical activity on same-day negative and positive affect. Specifically, stressor severity was associated with smaller elevation in daily negative affect on physically active days (b = 0.08, p < 0.001) compared to non-active days (b = 0.11, p < 0.001). Reductions in daily positive affect were greater on physically inactive days (b = -0.11, p < 0.001) compared to active days (b = -0.08, p < 0.001). These associations did not differ by age, but additional findings revealed that stressor severity was associated with greater elevation in negative affect among younger respondents (b = 0.12, p < 0.001) than older adults (b = 0.10, p < 0.001). These results highlight the importance of engagement in physical activity for emotional well-being under stressful situations in daily context.

